# Efficacy of Photorefractive Keratectomy vs. Topography-Guided Photorefractive Keratectomy for Refractive Errors and Aberrations Post-Penetrating Keratoplasty

**DOI:** 10.3390/jcm14041038

**Published:** 2025-02-07

**Authors:** Magdalena Kijonka, Anna Nowińska, Adam Wylęgała, Bogusława Orzechowska-Wylęgała, Bogdan Dugiełło, Katarzyna Kryszan, Edward Wylęgała

**Affiliations:** 1 Chair and Clinical Department of Ophthalmology, School of Medicine in Zabrze, Medical University of Silesia in Katowice, District Railway Hospital, 40-760 Katowice, Poland; anna.nowinska@sum.edu.pl (A.N.); adam.wylegala@gmail.com (A.W.); bogdan.dugiello@gmail.com (B.D.); kkryszann@gmail.com (K.K.);; 2 Department of Ophthalmology, District Railway Hospital in Katowice, 40-760 Katowice, Poland; 3 Health Promotion and Obesity Management, Pathophysiology Department, Medical University of Silesia in Katowice, 40-752 Katowice, Poland; 4 Department of Pediatric Otolaryngology, Head and Neck Surgery, Chair of Pediatric Surgery, Medical University of Silesia, 40-760 Katowice, Poland; borzechowska-wylegala@sum.edu.pl

**Keywords:** topography-guided, photorefractive keratectomy, penetrating keratoplasty, visual outcomes, refractive surgery, higher-order aberrations

## Abstract

**Background:** Managing post-keratoplasty astigmatism remains challenging. Even though graft viability is the main concern in keratoplasty, astigmatism might hinder vision recovery following a successful corneal transplant. Photorefractive keratectomy (PRK) and topography-guided PRK may be options for correcting refractive errors in patients who underwent keratoplasty. The aim of the study was to compare the results of PRK and topography-guided PRK in patients who had undergone corneal keratoplasty. **Methods:** This study was conducted at the Chair and Ophthalmology Clinic of the Medical University of Silesia, at the Railway Hospital Katowice, from 2023 to 2024. Patients who underwent penetrating keratoplasty due to keratoconus or corneal scar (post-traumatic and post-inflammatory) with a residual spherical myopic or mixed myopic astigmatic refractive defect were included in this study. The studied patients were divided into two groups: 15 patients underwent PRK and 15 patients underwent topography-guided PRK. Each participant underwent a preoperative examination, including uncorrected visual acuity (UCVA) and best spectacle-corrected visual acuity (BSCVA) measured on the Snellen chart (LCD panel for visual acuity testing Frey CP-400, Optotech Medical, Niepołomnice, Niepołomice, Poland), cycloplegic refraction, corneal pachymetry and topography (Schwind Sirius+, Pentacam), wavefront aberrations (Schwind Peramis, Pentacam), applanation tonometry, and anterior and posterior segment examinations, conducted at baseline and 1, 3, 6, and 12 months. **Results:** Keratoconus was the most common reason for keratoplasty (80% vs. 60%). Following PRK, the mean KI in group (1) decreased significantly to 43.88 ± 3.64 (*p* < 0.001), and in the group (2), the mean diopters decreased significantly after the intervention to 46.46 ± 2.80 (*p* < 0.001). The mean spherical refractive error in group (2) changed significantly after the intervention, reaching −2.72 ± 1.28 D (*p* < 0.001). The mean cylinder in group (1) changed significantly after PRK to have a mean of −2.75 ± 1.44 D (*p* < 0.001). Also, in group (2), it changed significantly after the intervention to have a mean of −2.95 ± 1.99 D (*p* < 0.001). There was a significant increase in both uncorrected visual acuity (UCVA) and best-corrected visual acuity (BCVA) after topography-guided PRK at all the follow-up points of 1, 3, 6, and 12 months (*p* < 0.001). The mean higher-order corneal aberrations (HOAs) after topography-guided PRK were 1.33 ± 0.50, ranging from 0.22 to 2.34 (*p* < 0.001). **Conclusions:** Topography-guided PRK is safer and more effective in correcting aberrations and refractive errors after corneal keratoplasty than regular PRK. Additionally, topography-guided PRK reduces high-order aberrations by utilizing advanced topographic mapping of the cornea, enabling precise customization of the treatment to address individual corneal surface irregularities.

## 1. Introduction

Keratoconus, a chronic non-inflammatory corneal disorder, causes myopia, irregular astigmatism, and distorted vision through progressive collagen alteration and corneal thinning [1]. The primary obstacle to proper visual rehabilitation in patients who have had penetrating keratoplasty is postoperative astigmatism, which can reach up to 20 diopters (D) and cause significant anisometropia. Numerous factors, including previous corneal thinning, eccentric trephination of the host, vascularization, preoperative keratoconus, graft size, donor eye astigmatism, and the length, depth, tension, and placement of corneal sutures, might result in excessive astigmatism. Unfortunately, using glasses for vision rehabilitation frequently leads to suboptimal visual outcomes [2].

In such cases, rigid gas-permeable contact lenses are often the preferred option despite their application challenges. However, they have been shown to increase the risk of transplant rejection by inducing peripheral neovascularization [3].

When traditional optical methods for eyesight rehabilitation do not provide sufficient results, these refractive defects are corrected through photorefractive keratectomy (PRK). Either topography-guided or wavefront-guided approaches are used for the process. In wavefront-guided therapies, the whole optical system provides the information in. However, corneal topography is used to collect data for topography-guided therapies, which are mostly employed for patients with irregular corneas [4].

Topography-guided PRP has been used for approximately ten years to treat irregular astigmatism and ametropia caused by keratoconus, with positive outcomes. Under the direction of a topographical map, this procedure is based on corneal tissue ablation fitted on an ideal corneal form, often a spherical form [5,6].

In addition to flattening some of the cone peaks, topography-guided photorefractive keratectomy also smooths a wider, arcuate portion of the cornea away from the cone, typically in the superior nasal periphery. This ablation pattern, similar to hyperopic treatment, results in some steepening, or elevation, next to the cone, which helps normalize and smooth the cornea. Nevertheless, this method may result in long-term issues such as worsening keratoconus, with increasing corneal thinning and apical opacity, which may lead to corneal perforation [7].

This study aims to compare the outcomes of PRK and TG-PRK in patients following penetrating keratoplasty. Both techniques—PRP and topography-guided PRK—represent significant advancements in the personalization of treatment in ophthalmology, resulting in improved outcomes, particularly in cases requiring more precise correction of refractive errors. These laser techniques contribute to improved postoperative functionality for patients following keratoplasty.

## 2. Patients and Methods

This study assessed the effectiveness of PRK in treating astigmatism and myopia. All the patients exhibited myopic astigmatism following keratoplasty and were intolerant of contact lenses and glasses. Before undergoing refractive surgery, all the patients had their sutures removed at least six months before and the stability of their refractive defect was verified following suture removal. Complete suture removal, steady refraction, and enough corneal thickness to guarantee a residual stromal thickness of at least 350 µm following PRK were prerequisites for inclusion.

The uncorrected visual acuity (UCVA), best spectacle-corrected visual acuity (BSCVA) measured on the Snellen chart, cycloplegic refraction, corneal pachymetry and topography (Schwind Sirius+, Pentacam), wavefront aberrations (Schwind Peramis, Pentacam), applanation tonometry, and anterior and posterior segment examinations were performed in the period of 1, 3, 6 and 12 months.

Every patient signed an informed consent form after being fully informed of the procedure’s dangers and advantages.

The patients were treated from June 2023 to December 2024. All the patients in both groups were of Caucasian ethnicity.

Inclusion criteria:

- Patients aged 38–50 after keratoplasty due to keratoconus or scar (post-traumatic or post-inflammatory) with residual spherical myopic or mixed myopic astigmatic refractive errors.

Exclusion criteria:

- Patients with cataract, glaucoma, amblyopia, uveitis, retinal diseases, active systemic diseases and a history of prior ophthalmic surgery were excluded from our study.
- Residual stromal thickness (RST) < 350 µm

The decision to perform either photorefractive keratectomy (PRK) or topography-guided photorefractive keratectomy (TG-PRK) was made based on the corneal parameters, refractive errors, higher-order aberrations, and results of individual optometric examinations. PRK was chosen when the cornea had healed symmetrically after penetrating keratoplasty, with no significant irregularities or higher-order aberrations. TG-PRK after penetrating keratoplasty was recommended in cases of corneal irregularities, high or irregular astigmatism, or the presence of higher-order aberrations that required correction. An additional consideration for TG-PRK was the corneal thickness, as the procedure typically requires more corneal tissue to be ablated compared to PRK. Residual stromal thickness of at least 350 µm was required after PRK or TG-PRK.

Technique: The Schwind Amaris 1050RS excimer laser (Schwind: Kleinostheim, Germany) was used to conduct the PRK and topography-guided PRK. All the procedures were performed by one surgeon. Before surgery, three drops of proxymetacaine were administered for corneal and conjunctival anesthesia. A blade was used to scrape off the epithelium. Data from the topography and aberrometry (Schwind Sirius+, Schwind Peramis) were used to plan the treatments, and the treatment data were transferred to the Schwind Amaris 1050RS excimer laser. After ablation, 0.02% mitomycin C (MMC) was administered for 30 s.

The refraction error (low-order aberration) or high-order aberration had to be reduced as much as feasible during the surgery, without inducing hyperopia.

Ablation zone: The optical zone was the same in all cases, dependent on the size of the transplanted corneal flap, and was set to 7.0 mm. The transition zone was automatically calculated by the laser based on the planned refractive correction.

A bandage soft contact lens was placed on the operated eye immediately after PRK.

Drops after laser: Levofloxacin, tobramycin and dexamethasone.

## 3. Statistical Analysis

Microsoft Excel 2016 for Windows, which is part of the Microsoft Office suite and was produced by Microsoft Corporation in the Redmond, Washington, United States, was used to gather, code, and enter data into a spreadsheet. IBM Statistical Package for Social Sciences software (SPSS), 25th version, IBM, United States, was used to analyze the data. The normality of the distribution was confirmed using the Kolmogorov–Smirnov test. Whereas categorical data were represented by numbers and percentages, continuous data were represented by the mean ± standard deviation, median, and IQR. Tables and graphs were used to display the data. When the *p*-value was less than or equal to 0.05, the results were deemed statistically significant; when it was less than or equal to 0.001, they were deemed highly statistically significant. The used tests were the following:

1. Chi-square test: Used to compare groups when analyzing categorical data.
2. When more than 20% of the cells have an anticipated count of less than 5, the chi-squared test was corrected using Fisher’s exact test or Monte Carlo adjustment.
3. Separate samples: When comparing two groups under study, the *t*-test was utilized for quantitative variables that were regularly distributed.
4. The Mann–Whitney test was used to compare two groups under study when quantitative data were abnormally distributed.
5. Repeated examples: The mean difference between two related groups was compared using the *t*-test.
6. Wilcoxon: Two related samples, matched samples, or a paired difference test of repeated measurements on a single sample were compared using the rank test to see whether their population mean ranks differed.
7. Friedman’s test, often known as the Fr test, was used to determine whether there was a significant difference between more than two dependent non-parametric data points over a range of time intervals in continuous data.

## 4. Results

This study was conducted on 30 patients who underwent corneal keratoplasty. The studied patients were divided into two groups: 15 patients underwent PRK and 15 patients underwent topography-guided PRK.

The mean age in group (1) and group (2) was 43.33 ± 2.74 years and 44.60 ± 2.20 years, respectively. No significant difference was observed between the two groups regarding age (*p* > 0.05) (Table 1).

In group (1), there were nine men (60.0%) and six women (40.0%). In group (2), there were eight men (53.3%) and seven women (46.7%). There was no significant difference between the groups regarding gender (*p* > 0.05).

Keratoconus was the most frequent cause of keratoplasty, rather than a scar, in group (1) and group (2) (80% vs. 60%). There was no significant difference between the two groups regarding the cause of keratoplasty (*p* > 0.05).

Also, there was no difference in pachymetry, K1, and K2 between the two groups before treatment.

Regarding the operative data, there was no difference between the two groups regarding the ablation depth and residual stromal tissue (*p* > 0.05).

After the intervention, K1 and K2 were significantly lower in group (1) compared to group (2) (*p* = 0.038 and *p* = 0.001, respectively). Furthermore, a significant decline in K1 and K2 after the intervention compared to their level before the intervention was observed in both group (1) (*p* < 0.001) and group (2) (*p* < 0.001) (Table 1).

On the other hand, there was a significant difference between group (1) and group (2) as regards the spherical equivalent, which was significantly decreased in group (1) compared to group (2) before (*p* < 0.001) and after (*p* = 0.026) treatment (Table 1).

There was no difference between group (1) and group (2) regarding UCVA and BCVA pre-PRK and at 1, 3, 6, and 12 months postoperatively (*p* > 0.05). In the longitudinal analysis, in the comparison between UCVA and BCVA, there was a significant improvement in visual acuity before and after 1 and 3 months of treatment. However, there was an insignificant difference 6 and 12 months after treatment as they became nearly the same (*p* > 0.05), which indicated good correction of the VA in the two groups at the long-life period (Table 2).

Two cases (13.3%) in group (1) had complications (one case had delayed re-epithelialization, and in one case, double vision was caused by exotropia). One case had complications in group (2), which caused rejection. There was no significant difference between group (1) and group (2) regarding complications (*p* > 0.05).

A significant decline was observed in the total aberrations and higher-order corneal aberrations (HOAs) postoperatively compared to their readings preoperatively in the TG-PRK group (*p* < 0.001). However, a significant improvement was noticed in the vertical coma postoperatively compared to its reading preoperatively in the TG-PRK group (*p* = 0.003) (Table 3).

## 5. Discussion

In order to evaluate the outcomes of PRK versus topography-guided PRK in patients who had undergone corneal keratoplasty, this research was conducted. Thirty individuals who had undergone corneal keratoplasty participated in this study. The patients in this study were split into two groups: 15 had TG-PRK and 15 had photorefractive keratectomy.

Groups (1) and (2) in our study had mean ages of 43.33 ± 2.74 and 44.60 ± 2.20 years, respectively. In groups (1) and (2), keratoconus was the most common reason for keratoplasty (80% vs. 60%), and there was no discernible difference in the two groups’ ages or reasons for having keratoplasty.

In our study, patients over 60 years of age did not decide to undergo treatment, explaining their decision with lower visual requirements compared to younger patients. This age group had reduced expectations regarding the quality of their vision. They perceived it as natural and not as a problem requiring treatment.

Similarly, Colombo-Barboza et al. [8] assessed 15 patients (30 eyes) with a mean age of 55.1 ± 3.5 years who had undergone topography-guided PRK for uneven astigmatism due to radial keratotomy. Tambe et al. [9] further noted in their case report that the median age of patients exhibiting keratoconus development at the time of PRK was around 47. Performing a PRK treatment, which causes more corneal thinning, may be a trigger for the disease’s advancement in elderly individuals because it is quite uncommon to observe progression at this advanced age.

However, in Nooreldin’s [2] study, the patients’ ages varied from 23 to 35, with a mean of 28.6 ± 3.7. Also, a study by Bilgihan et al. [10] showed that 16 people, whose mean age was 28.4 ± 6.8 years, had their post-PKP myopia and astigmatism well treated with PRK in 16 eyes (eight right and eight left).

The preoperative flat keratometry value (K1) in group (1) in the current study varied from 44.4 to 56 D, with a mean of 49.14 ± 3.62 D. Following PRK, the mean K1 decreased considerably to 43.88 ± 3.64 (*p* < 0.001). In group (2), the preoperative value varied between 43 and 44.55 D, with a mean of 48.31 ± 3.09 D. Following topography-guided PRK, the mean diopters decreased considerably to 46.46 ± 2.80 (*p* < 0.001). Similar to this, group (1)’s preoperative steep keratometry (K2) values varied from 51.45 to 58.16 D, with a median of 53.80 D; following PRK, these values considerably fell to 45.72 diopters (*p* < 0.001). Additionally, in group (1), the preoperative value had a median of 53 D and varied from 51.25 to 60.0 D; this was dramatically reduced following topography-guided PRK, which had medians of 48.17 diopters (*p* < 0.001).

Sakla et al. [11] conducted research on 85 eyes with a mean 12-month follow-up. Additionally, they demonstrated an improvement in the keratometric readings. Nooreldin [2] showed that the preoperative flat keratometry value (K1) had a median of 42 D and ranged from 41.5 to 43.5 D. It decreased considerably to medians of 36, 35.5, and 35 D at 1, 2, and 3 months after PRK (*p* < 0.001). Similar to this, the preoperative value for steep keratometry (K2) varied from 45.25 to 48.25 D, with a median of 47 D. After 1, 2, and 3 months, the medians dropped dramatically to 40.13, 40, and 40 diopters, respectively (*p* < 0.001). They examined the relationship between the postoperative measures and discovered that K1 and K2 were comparable between 1 and 2 months and between the 2- and 3-month assessments but significantly reduced after 3 months compared to 1 month (*p* = 0.002 and 0.001 for K1 and K2, respectively).

Comparing surface ablation to other refractive operations, prior research and fundamental science have shown that it is more effective at preserving the cornea’s mechanical characteristics and hysteresis [12,13,14]. This is explained by the formation of a second fibrocellular layer in the ablated corneal layers, which strengthens the cornea and serves as a barrier to stop the keratoconus from worsening [15]. Although there is still potential, these basic adjustments might reduce the likelihood of haze [12].

Our study’s results have revealed that there was no significant difference between groups (1) and (2) after PRK regarding the UCVA, as the median UCVA was (*p* > 0.05) during all of the preoperative period, and at 1, 3, 6, and 12 months after PRK, while there was a significant increase in the mean UCVA and BCVA after 1 and 3 months post-PRK; however, there was no difference between BCVA and UCVA at 6 and 12 months postoperatively. This indicated that both techniques showed VA improvement in the long-life period.

Nooreldin [2] revealed that the BCVA of the examined eyes varied between 0.01 and 0.2, with a median value of 0.1 and an IQR between 0.1 and 0.2 prior to surgery. After a month, this significantly improved to a median value of 0.5, with an IQR between 0.4 and 0.58 (*p* = 0.003). After two and three months post-PRK, it further improved to a median of 0.7, with an IQR between 0.7 and 0.8 (*p* < 0.001), and all the eyes had a BCVA of 0.8 after three months.

Similar research was conducted by Al-Sahafi and Aljindan [16] to assess the PRK outcomes in patients who had keratoplasty using wavefront topography-guided therapeutic approaches. They found that both therapies produced positive outcomes for BCVA following PRK. A study by Huang et al. [17] showed that the SE had decreased and the UCVA had significantly improved. In our clinic, we frequently removed the suture a year after keratoplasty; this disparity may have affected the results concerning the UCVA and BCVA. They maintained the suture in place until it broke or became weak in order to minimize refractive errors.

Another study conducted by Laíns et al. [18] investigated the efficacy of TG-PRK in curing the refractive deficits in 31 eyes after keratoplasty (23 of which were diagnosed with keratoconus). The refractive parameters demonstrated a significant improvement, and 96.8% (n = 30) of the eyes displayed a gain of >1 UCDVA line.

Ward et al. [19] evaluated the efficacy of adjuvant MMC treatment in combination with PRK in patients who underwent 20 eye procedures. Ten eyes (50%) had two or more lines of BCVA, eighteen eyes (90%) had a BCVA of 20/40 or greater, and thirteen patients (65%) had a UDVA of 20/40 or higher. Dos Santos Forseto et al. [4] showed that uncorrected visual acuity of at least 20/40 was present in 19 out of 36 eyes (52.8%). A UDVA of 20/40 or greater was present in 35 out of 79 eyes (44%) and a BCVA of 20/40 or better in 67 out of 79 eyes (85%) in the research conducted by Al-Sahafi and Aljindan [16]. Furthermore, in their trial, Kovoor et al. [20] showed that 8 out of 14 patients who received PRK therapy saw an improvement in their uncorrected visual acuity of at least two lines.

Our study’s results have revealed that a significant decline was observed in the total aberrations and HOA postoperatively compared to their readings preoperatively in group (2). The mean total aberrations before TG-PRK were (4.73 ± 2.18), ranging from 2.10 to 10.20, while after TG-PRK, the mean was (1.95 ± 1.04), ranging from 0.96 to 5.10 (*p* < 0.001). Also, the mean HOA before TG-PRK was (1.73 ± 0.64), ranging from 0.32 to 2.86, while after TG-PRK, the mean was (1.33 ± 0.50), ranging from 0.22 to 2.34 (*p* < 0.001).

In line with our findings, De Rosa et al. [5] found that aberrometry showed a reduction in the total aberrations’ root mean square, while a statistically significant difference in the total RMS was found one year after treatment (*p* = 0.027, paired Student’s *t*-test). They did not observe a statistically significant change in the spherical aberration and coma at 12 months after surgery.

A reduction in the total aberrations leads to an improvement in the quality of the corneal front surface and so to a better image optical quality [21].

## 6. Limitations of This Study

Our study was limited by the following factors:

- Small sample size: This study included a small group of patients, which allowed for a more detailed analysis of individual cases, potentially leading to more precise conclusions. This approach, while limited in terms of generalizability, contributes valuable data to an area where evidence is lacking. Further, larger studies will be planned based on the findings of this study.
- Lack of G-power analysis.
- Follow-up period: The follow-up period was tailored to the aims of this study, which focused on gathering sufficient data to compare laser correction techniques.
- Lack of adjustments for multiple comparisons (such as the Bonferroni correction).

## 7. Conclusions

1. Simultaneous topography-guided PRK is a safe and effective technique, offering better outcomes than regular PRK in correcting refractive errors and aberrations after PKP.
2. Topography-guided PRK significantly reduces astigmatism to subclinical levels and improves higher-order aberrations, enhancing visual quality and contrast sensitivity.
3. Future studies with larger samples and longer follow-ups are needed to confirm these findings and assess the long-term effectiveness.

In summary, a comparison of PRK and topography-guided PRK reveals that the topography-guided technique provides significant improvements in treatment accuracy and quality, especially in patients with irregular corneas or high-order aberrations. Clinicians should consider topography-guided PRK in cases where traditional PRK may not offer optimal results, particularly in patients following penetrating keratoplasty.

## Figures and Tables

**Table 1:** Patients’ characteristics and ocular operative parameters before and after treatment.

Groups	PRK	TG-PRK	Significance	
	Mean ± SD	Mean ± SD	t	*p*
Age (years)	43.3 ± 2.74	44.6 ± 2.20	1.396	0.174
Pachymetry (µm)	585.07 ± 20.96	585.0 ± 36.62	0.589	0.926
Ablation depth without Ep. (µm)	138.4 ± 3.72	134.4 ± 15.66	0.354	0.723
Residual stromal thickness (µm)	391.67 ± 21.45	395.6 ± 34.67	0.374	0.712
Spherical equivalent (D)				
- Before treatment	−11.6 ± 1.92	−7.95 ± 1.08	7.31	0.000 **
- After treatment	−1.25 ± 0.25	0.75 ± 0.12	2.36	0.026 *
Paired *t*-test	19.84	12.75		
Significance (*p*)	0.000 **	0.000 **		
K1 (D)				
- Before treatment	49.14 ±3.62	48.31 ± 3.09	0.681	0.501
- After treatment	34.88 ±3.64	36.46 ± 2.80	2.174	0.038 *
Paired *t*-test	9.751	4.743		
Significance (*p*)	0.000 **	0.001 **		
K2 (D)				
- Before treatment	53.80 ± 4.28	53.0 ± 4.31	0.291	0.771
- After treatment	41.72 ± 3.92	44.17 ± 3.68	3.195	0.001 **
Paired *t*-test	5.951	4.743		
Significance (*p*)	0.012 *	0.018 *		

t: paired *t*-test, * *p* < 0.05: significant, ** *p* < 0.001: highly significant, SD: standard deviation, D: diopter, PRK: photorefractive keratectomy, TG-PRK: topographic guided PRK, Ep.: epithelium.

**Table 2:** Comparison of the visual acuities between the two groups before and after treatment.

Visual Acuity (Decimal Values)	PRK (n = 15)		TG-PRK (n = 15)		*p* Value (BCVA by *t*-Test)
	Range	Mean ± SD	Range	Mean ± SD	
UCVA pre-PRK	0.01–0.01	0.01 ± 0.0	0.01–0.10	0.027 ± 0.025	0.755
BCVA pre-PRK	0.10–0.50	0.25 ± 0.14	0.10–0.50	0.233 ± 0.11	
*p* value		0.000 **		0.000 **	
UCVA post-1 m	0.01–0.01	0.11 ± 0.0	0.10–0.30	0.15 ± 0.08	0.262
BCVA post-1 m	0.10–0.50	0.29 ± 0.11	0.10–0.50	0.32 ± 0.09	
*p* value		0.001 **		0.001 **	
UCVA post-3 m	0.01–0.01	0.21 ± 0.0	0.10–0.30	0.25 ± 0.07	0.124
BCVA post-3 m	0.10–0.50	0.29 ± 0.12	0.10–0.50	0.34 ± 0.09	
*p* value		0.001 **		0.037 *	
UCVA post-6 m	0.01–0.01	0.25 ± 0.0	0.10–0.30	0.28 ± 0.06	0.225
BCVA post-6 m	0.10–0.50	0.29 ± 0.11	0.10–0.50	0.33 ± 0.08	
*p* value		0.257		0.145	
UCVA post-12 m	0.01–0.01	0.24 ± 0.0	0.10–0.30	0.31 ± 0.06	0.225
BCVA post-12 m	0.10–0.50	0.30 ± 0.11	0.10–0.50	0.33 ± 0.08	
*p* value		0.256		0.324	

t: unpaired *t*-test, * *p* < 0.05: significant, ** *p* < 0.001: highly significant, m: month, post: post-treatment period.

**Table 3:** Aberrations before and after intervention in group (2).

Aberration	Before TG-PRK		After TG-PRK		Significance	
	Mean	±SD	Mean	±SD	r	*p*-Value
Total	4.73	±2.18	1.95	±1.04	7.882	<0.001 **
HOA	1.73	±0.64	1.33	±0.50	5.657	<0.001 **
Trefoil vertical	0.16	±0.80	0.03	±0.30	0.957	0.355
Coma vertical um	−1.20	±1.05	−0.87	±0.73	−3.626	0.003 **
Coma horizontal	0.25	±0.48	0.19	±0.36	1.878	0.081
Trefoil horizontal	0.17	±0.87	0.08	±0.36	0.651	0.526
Tetrafoil oblique	−0.11	±0.42	−0.10	±0.30	−0.080	0.937
Astigmatism oblique	−0.17	±0.40	−0.14	±0.27	−0.927	0.369
Spherical aberration	−0.06	±1.02	−0.11	±0.71	0.482	0.637
Astigmatism cardinal	−0.15	±0.33	−0.10	±0.20	−1.396	0.184
Tetrafoil cardinal	0.01	±0.30	−0.01	±0.13	0.415	0.684

*p* value > 0.05: not significant, ** *p* ˂ 0.01: highly significant, HOA: high-order aberration.

## Data Availability

Data are available on request due to restrictions (e.g., privacy or ethics).
